# Hierarchical microstructure strengthening in a single crystal high entropy superalloy

**DOI:** 10.1038/s41598-020-69257-8

**Published:** 2020-07-22

**Authors:** Yung-Ta Chen, Yao-Jen Chang, Hideyuki Murakami, Taisuke Sasaki, Kazuhiro Hono, Chen-Wei Li, Koji Kakehi, Jien-Wei Yeh, An-Chou Yeh

**Affiliations:** 1 Department of Materials Science and Engineering, National Tsing Hua University, 101, Sec. 2, Kuang-Fu Road, Hsinchu, 30013 Taiwan, ROC; 2 Research Center for Structural Materials, National Institute for Materials Science, 1-2-1 Sengen, Tsukuba, 305-0047 Japan; 3 High Entropy Materials Center, National Tsing Hua University, 101, Sec. 2, Kuang-Fu Road, Hsinchu, 30013 Taiwan, ROC; 4 Department of Nanoscience and Nanoengineering, Waseda University, 3-4-1 Okubo, Shinjuku, Tokyo, 169-8555 Japan; 5 Research Center for Magnetic and Spintronic Materials, National Institute for Materials Science, 1-2-1 Sengen, Tsukuba, 305-0047 Japan; 6 Department of Mechanical Engineering, Tokyo Metropolitan University, 1-1 Minami-osawa, Hachioji-shi, Tokyo, 192-0397 Japan

**Keywords:** Mechanical properties, Metals and alloys

## Abstract

A hierarchical microstructure strengthened high entropy superalloy (HESA) with superior cost specific yield strength from room temperature up to 1,023 K is presented. By phase transformation pathway through metastability, HESA possesses a hierarchical microstructure containing a dispersion of nano size disordered FCC particles inside ordered L1_2_ precipitates that are within the FCC matrix. The average tensile yield strength of HESA from room temperature to 1,023 K could be 120 MPa higher than that of advanced single crystal superalloy, while HESA could still exhibit an elongation greater than 20%. Furthermore, the cost specific yield strength of HESA can be 8 times that of some superalloys. A template for lighter, stronger, cheaper, and more ductile high temperature alloy is proposed.

## Introduction

The development of high-entropy alloys (HEAs) has broken through the frame of conventional alloys by exploring the vast composition space of multi-principle elements^1–6^, and their extraordinary mechanical properties have been a subject of interest, for examples, single-phase CoCrFeMnNi HEA showed high tensile strength of 1,280 MPa with elongation up to 71% at cryogenic temperature^7^; the compressive strength could reach 2,240 MPa at 298 K for Al_0.5_CoCrFe_0.5_NiTi_0.5_ HEA^8^ and 1,520 MPa at 873 K for Al_0.5_CrNbTi_2_V_0.5_ HEA^9^ due to the presence of intermetallic phases, such as σ^8^, B2^8^ and Laves^9^. Most of the high temperature mechanical properties data of HEAs in literatures are from compression tests^3,4,10^. Although some studies have reported tensile tested data of HEAs, there are only a few at elevated temperatures^3,4,10–17^. The tensile strength of HEA could be degraded severely at elevated temperatures^4,17,18^, for example at 1,023 K, the tensile yield strength of CoCrFeMnNi was lower than 100 MPa^17^. In conventional precipitation strengthened superalloys, precipitation of coherent Ni_3_(Al, Ti) L1_2_ structured phase in FCC matrix can provide effective strengthening^19^. Recent studies have shown that coherent L1_2_ phase could also be an effective strengthener in HEAs^11,20–22^, for example, L1_2_ precipitation in Al_7_ (Fe, Co, Ni)_86_Ti_7_ resulted a combination of high tensile yield strength (1,028 MPa) with large elongation (47.8%) at room temperature^20^. However, the elevated temperature tensile strength of HEA could be limited by insufficient fractions and relatively low solvus of strengthening phases in HEAs^11^. One of the highest reported tensile yield strength of HEAs at 1,023 K was 473 MPa for cast-type Al_10_Co_25_Cr_8_Fe_15_Ni_36_Ti_6_, which contained a FCC matrix with 46% L1_2_ and 5% B2 phases by volume fractions^23^, although its yield strength could surpass those of solid solution type superalloys such as 800H and Inconel617, it was weaker than advanced precipitation strengthened cast-type superalloys^24^. Alloy design for higher L1_2_ phase fraction and solvus temperature are required to further improve the high temperature tensile strength of HEAs. However, the high entropy composition scope^3,4^ of 5.0 at.% ≤ x ≤ 35.0 at.% could jeopardize the thermal stability of ordered phase such as L1_2_ phase^25–28^, and Ni-rich HEA with Ni content beyond 35 at.% has provided a window of opportunity to design thermally stable L1_2_ precipitation in HEA while retaining the compositional configurational entropy |ΔS_conf._|> 1.5 R, where R is the universal gas constant^3,4^; this class of HEA has been named High Entropy Superalloys (HESA)^25–29^. Recently, the concept of HESA has been adopted by Zhang et al.^29^, Ni_45 − x_(Fe, Co, Cr)_40_(Al, Ti)_15_Hf_x_ based alloys were studied in as-cast condition; the tensile strength of these HESAs could reach 960 MPa at 1,023 K.

In this work, the HESA (HESA-3^27^) of interest is shown in Table 1; it is a cast-type alloy with a density of 7.96 g/cm^3^, comparing to advanced cast superalloys such as CMSX-4^30^, the raw materials cost of this HESA was 84% cheaper due to the absence of Re element content. This HESA in directionally solidified form^27^ had been reported to possess a tensile yield strength of 855 MPa at 1,023 K, which was slightly lower than that of CMSX-4 prepared by the standard process^24^. This work demonstrates that phase transformation pathway through metastability can induce an interesting hierarchical microstructure, which can further increase the elevated temperature tensile yield strength of HESA. The aim of this article is to present a microstructure template for developing future advanced high temperature alloys with improved cost-performance.

**Table 1:** Chemical compositions of HESA and different phases analyzed by APT.

HESA (at.%)	Al	Ti	Nb	Ni	Co	Cr	Fe	Mo	W	ΔS_conf._ (-R)
Nominal	**10.2**	**5.8**	**1.2**	**48.3**	**16.9**	**7.4**	**8.9**	**0.9**	**0.4**	**1.58**
**HT-1 heat treatment (1,500 K 20 h /air cooling)**										
HE FCC matrix	**5.1** ± 0.03	**1.2** ± 0.02	**0.3** ± 0.01	**29.9** ± 0.07	**24.1** ± 0.06	**18.4** ± 0.05	**19.2** ± 0.06	**1.5** ± 0.02	**0.3** ± 0.01	**1.63**
HE FCC particles	–	–	–	–	–	–	–	–	–	–
ME L1_2_ phase	**13.1** ± 0.06	**8.3** ± 0.05	**1.3** ± 0.02	**56.6** ± 0.09	**13.2** ± 0.06	**2.3** ± 0.02	**4.5** ± 0.04	**0.5** ± 0.01	**0.2** ± 0.01	**1.38**
ME 2nd L1_2_ particles	**14.5** ± 0.69	**8.6** ± 0.41	**0.7** ± 0.18	**60.6** ± 0.87	**12.1** ± 0.57	**0.8** ± 0.21	**1.7** ± 0.31	**0.8** ± 0.20	**0.2** ± 0.10	**1.24**
**HT-2 heat treatment (1,500 K 20 h/air cooling + 1,023 K 20 h /water quenching)**										
HE FCC matrix	**3.5** ± 0.03	**0.8** ± 0.02	**0.1** ± 0.01	**23.9** ± 0.08	**27.5** ± 0.09	**20.4** ± 0.08	**21.7** ± 0.08	**1.6** ± 0.01	**0.5** ± 0.01	**1.61**
HE FCC particles	**2.6** ± 0.24	**0.4** ± 0.10	**0.1** ± 0.04	**22.8** ± 0.57	**28.0** ± 0.56	**21.7** ± 0.51	**23.2** ± 0.53	**0.9** ± 0.13	**0.3** ± 0.06	**1.55**
ME L1_2_ phase	**13.2** ± 0.08	**8.5** ± 0.06	**1.2** ± 0.02	**58.3** ± 0.11	**12.3** ± 0.07	**1.8** ± 0.02	**3.9** ± 0.04	**0.5** ± 0.02	**0.3** ± 0.01	**1.34**
ME 2nd L1_2_ particles	**14.6** ± 0.40	**8.9** ± 0.35	**0.6** ± 0.09	**61.5** ± 0.63	**10.6** ± 0.37	**0.6** ± 0.07	**1.7** ± 0.18	**1.0** ± 0.10	**0.5** ± 0.08	**1.24**

## Results

### Hierarchical microstructure of HESA

**Figure 1:**
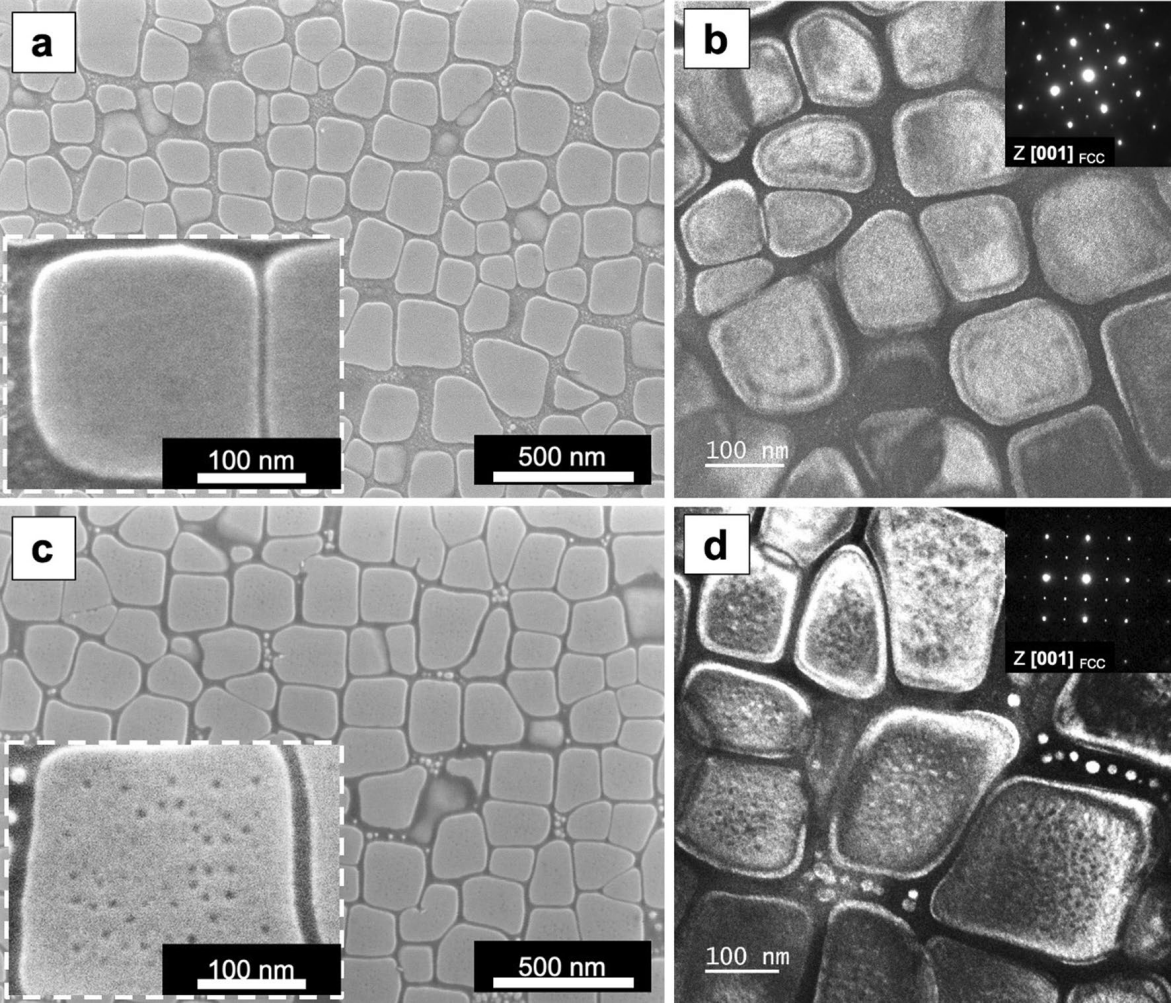
SEM and TEM observations of HESA after heat treatments. (**a**) SEM images of HT-1 sample, (**b**) TEM dark field image of HT-1 sample from L1_2_ (001) superlattice spot on [001] zone axis, (**c**) SEM images of HT-2 sample, and (**d**) TEM dark field image of HT-2 sample from L1_2_ (001) superlattice spot on [001] zone axis.

Single crystal samples of HESA, with the composition shown in Table 1, were heat treated by two different heat treatment steps, i.e. HT-1 and HT-2; Fig. 1 shows the heat treated microstructures. HT-1 consisted a single ramp from room temperature to 1,500 K in 20 h followed by air cooling, and the microstructure contained cuboidal precipitates and nano-particles, Fig. 1a, superlattice diffraction pattern observed on the [001] zone axis indicates that these precipitates possessed L1_2_ structure; the TEM dark field image taken from the L1_2_ (001) superlattice spot is shown in Fig. 1b. The precipitation of L1_2_ phase (143 nm in size) and secondary L1_2_ particles (1 nm in size) occurred during the air cooling process in HT-1, since the solvus of L1_2_ phase in this HESA was 1,472 K^27^. After an additional heat treatment at 1,023 K for 20 h followed by water quenching, the HT-2 process caused the L1_2_ phase to grow from 143 to 153 nm in average and secondary L1_2_ particles to coarsen into an average of 14 nm as shown in Fig. 1c; Furthermore, there were nano size particles appeared inside the cuboidal L1_2_ precipitates. Figure 1d shows the selected area diffraction pattern from the L1_2_ phase on the [001] zone axis together with the dark field TEM image taken from the L1_2_ (001) superlattice spot. Since L1_2_ precipitate has ordered FCC structure with the lattice constant close to the disordered FCC phases, one way to identify L1_2_ phase was by the L1_2_ superlattice spots, which could be used in dark field image to enhance the contrast between L1_2_ phases (brighter contrast) and the FCC matrix and nano-particles (darker contrast), Fig. 1d. Since the diffraction patterns exhibited only FCC and L1_2_ diffraction spots, so these nano-particles were very likely to possess disordered FCC structure.

**Figure 2:**
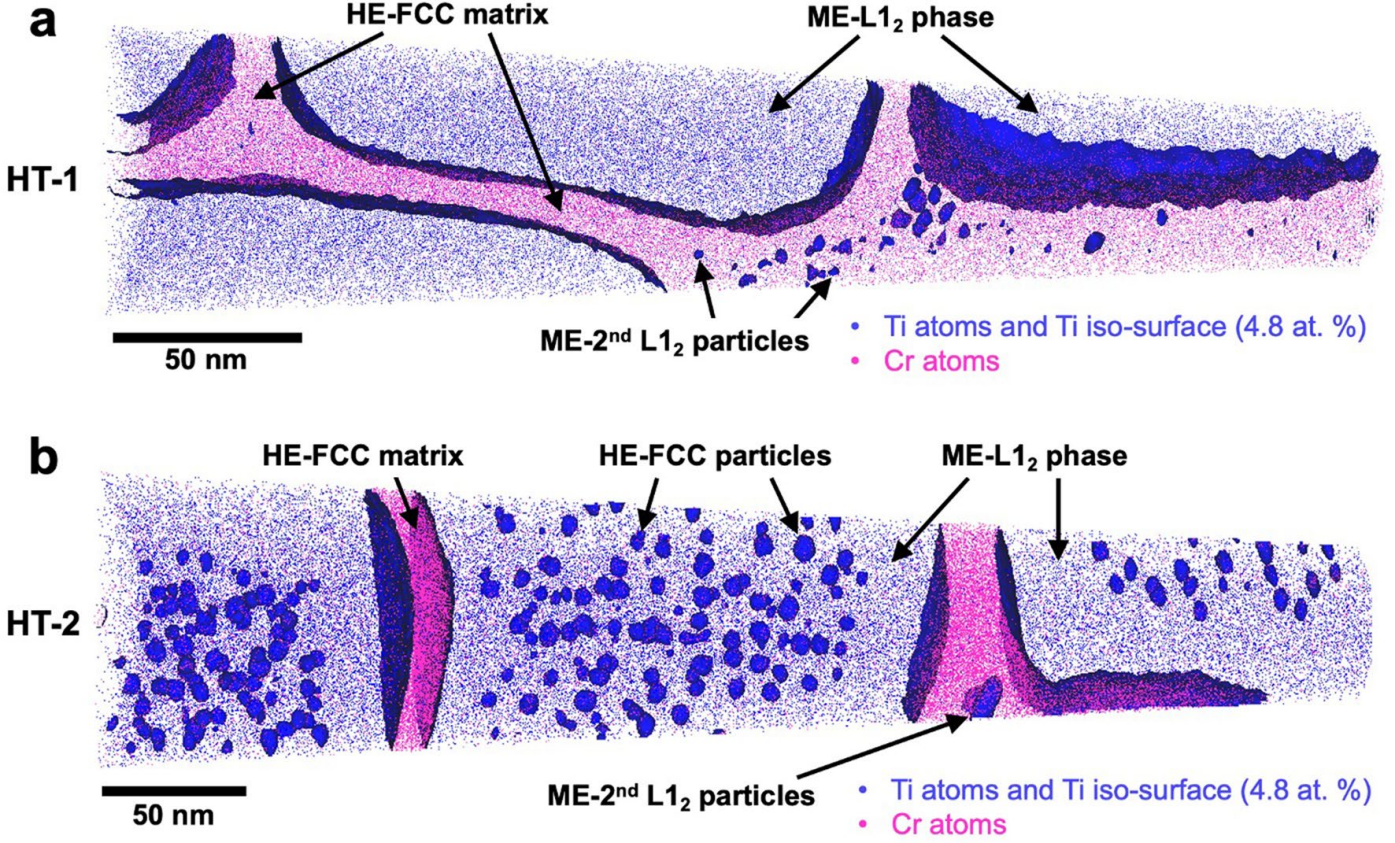
APT reconstructions of HESA. In the APT reconstructions of (**a**) HT-1 and (**b**) HT-2 states, Cr and Ti atoms are displayed for the clarity, and Ti 4.8 at.% is set as iso-surface.

Phases in HESA after HT-1 and HT-2 states have also been revealed by atom probe tomography reconstructions as shown in Fig. 2. To distinguish between FCC matrix and L1_2_ precipitates, Cr and Ti atoms have been depicted as these elements strongly partitioned to the FCC matrix and the L1_2_ precipitates, respectively. Ti 4.8 at.% iso-surface is set to highlight the FCC matrix—L1_2_ precipitate interface for clear visualization. In Fig. 2a, HT-1 state shows an FCC matrix with L1_2_ precipitates and secondary L1_2_ particles in the FCC matrix channel. The chemical composition of each phase was measured and listed in Table 1. In addition, partitioning coefficient which is defined as the concentration of element in FCC matrix divided by the concentration of the same element in the L1_2_ phase, can be calculated. In the HT-1 state, the partitioning coefficients of Al (0.39), Ti (0.14), Nb (0.23) and Ni (0.53) are below unity and that indicates a preferential partitioning behavior toward the L1_2_ phase; the partitioning coefficients of Co (1.83), Cr (8), Fe (4.27), Mo (3) and W (1.5) are greater than unity, suggesting that these elements preferred to partition to the FCC matrix; the elemental partitioning behaviors were similar to those reported in superalloys^19,31^. In Table 1, the magnitude of the configuration entropy (ΔS_conf._) for the FCC matrix is calculated to be 1.63 R, which agrees with the high-entropy composition definition. So, in the HT-1 condition, HESA possessed a High Entropy (HE) FCC matrix and Medium Entropy (ME) precipitates (L1_2_).

By contrast, the APT reconstructions in Fig. 2b shows an interesting hierarchical microstructure, in addition to the FCC matrix and the L1_2_ phases, there were nano-particles dispersion within the L1_2_ phase. These nano-particles were only present in the HT-2 state, not in the previous HT-1 state. Compositions of each phase in HT-2 state are summarized in Table 1. The calculated partitioning coefficients of Al, Ti, Nb, and Ni are 0.27, 0.09, 0.08 and 0.41, respectively; Co, Cr, Fe, Mo, and W are 2.24, 11.33, 5.56, 3.2, and 1.67, respectively. Partitioning coefficients in HT-1 and HT-2 states are similar. The nano-particles in the L1_2_ phase of HT-2 state possessed similar compositions as that of the FCC matrix, Table 1. According to the TEM analysis on structure (Fig. 1d) and APT composition measurement (Table 1), these nano-particles could be determined indirectly as disordered FCC particles. The magnitude of configuration entropy (ΔS_conf._) for the FCC matrix and the FCC particles are 1.61 R and 1.55 R, respectively, and both satisfy the high-entropy composition definition. Therefore, microstructure in HT-2 state contained High Entropy FCC matrix with a dispersion of Medium Entropy L1_2_ phase that also possessed nano-sized High Entropy FCC particles. Elemental partitioning behaviors and the concentration profiles evolution between the FCC and L1_2_ phases from HT-1 to HT-2 are shown in the Supplementary Fig. S1a,b.

The composition analysis of each phase in Table 1 have been confirmed by the lever rule analysis^26,27,31^, which is based on the principle of mass conservation. Plots of C_L12_–C_FCC_ versus C_n_–C_FCC_ from the HT-1 (Supplementary Fig. S2a) and HT-2 (Supplementary Fig. S2b) conditions can be obtained, where C_L12_, C_FCC_ and C_n_ represent the chemical compositions of ME-L1_2_ phase, HE-FCC matrix and bulk alloy, respectively. Line fittings could be conducted to verify the measured compositions, since the slopes could represent the mole fraction of the L1_2_ phase. The linear fitting slopes in Supplementary Fig. S2a,b correspond to 68.4% and 70.6% of L1_2_ precipitates in mole fractions, respectively; these values match well with the volume fractions of phases determined in Fig. 1.

### Thermodynamic simulation and phase evolution

**Figure 3:**
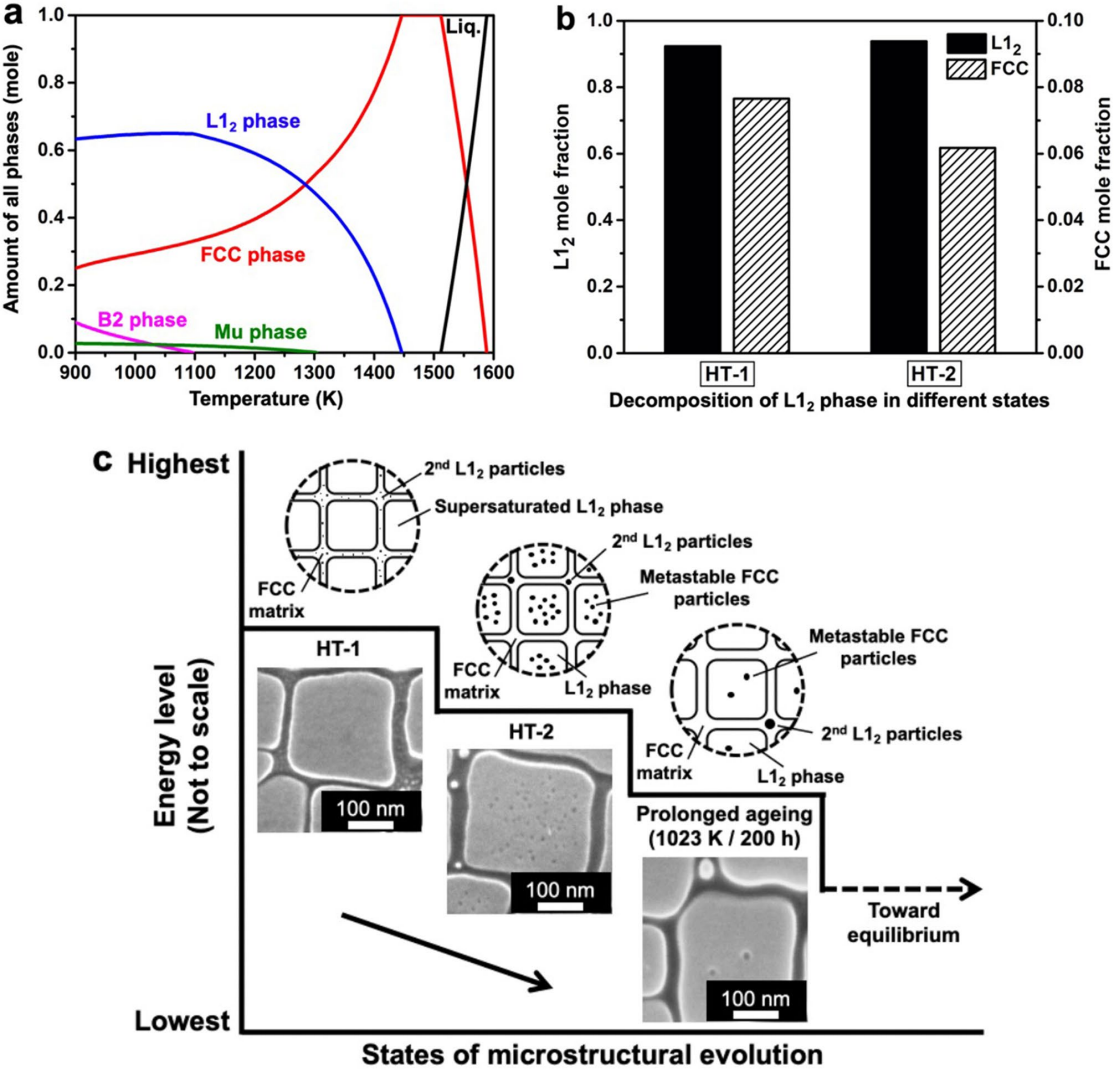
Thermodynamic simulation and experimental microstructure evolution of HESA. (**a**) ThermoCalc phase diagram based on HESA nominal composition, (**b**) ThermoCalc simulated decomposition of L1_2_ phases at 1,023 K based on the HT-1 and HT-2 ME-L1_2_ phase composition from APT data. (**c**) Summary of energy level to the microstructural evolution in HESA.

To elucidate the possible underlying mechanisms for the formation of the HE-FCC particles in the HT-2 condition, CALPHAD-based simulation, ThermoCalc^32^ (TCHEA3 databases) was used to predict phase diagrams of HESA and ME-L1_2_ phase in both HT-1 and HT-2 conditions, Fig. 3a,b. FCC and L1_2_ are the main phases predicted by the phase diagram in Fig. 3a; although minor B2 and Mu phases were also predicted, they were not found in the microstructure experimentally. Interestingly, CALPHAD phase fraction predictions (Fig. 3b) indicate that both ME-L1_2_ phase in HT-1 and HT-2 conditions could decompose into FCC and L1_2_ equilibrium phases, these results suggest the tendency to form FCC-structured particle inside the L1_2_ phase. However, the microstructure of the ME-L1_2_ phase in HT-1 state did not contain FCC particles. So, it can be deduced that the ME-L1_2_ phase in HT-1 condition was in a supersaturated state, Figs. 1a and 2a. On the other hand, the HT-2 heat treatment could promote the formation of FCC particles within ME-L1_2_ phase, Figs. 1c and 2b. To further clarify the supersaturation and decomposition of ME-L1_2_ phase, ThermoCalc was applied to determine the equilibrium phase compositions at 1,023 K, and the calculated partition coefficients are Al (0.19), Ti (0.02), Nb (0.12), Ni (0.41), Co (2.16), Cr (22.78), Fe (9.13), Mo (11.5) and W (11.0). Detailed equilibrium phase compositions are given in Supplementary Table S1. By comparing equilibrium elemental partitioning coefficients with those of the HT-1 and HT-2 states in Table 1, the HT-2 condition appeared to be closer to the equilibrium state than that of the HT-1 condition. The phase fractions calculated from the ME-L1_2_ phase composition of HT-1 state indicate that the L1_2_ phase could decompose into 7.7 mol% FCC phase at 1,023 K (Fig. 3b). By contrast, FCC phase fraction at 1,023 K calculated from the HT-2 composition was 6.2 mol% (Fig. 3b); the actual fraction of HE-FCC particles measured experimentally was 4.3 vol. % in the L1_2_ phase, indicating that the HT-2 state was still not at the equilibrium state. Therefore, the formation of FCC particles in HESA was driven by the supersaturation of FCC phase formers inside the L1_2_ phase.

The microstructural evolutions of HESA from HT-1 to HT-2 and prolonged ageing are illustrated in Fig. 3c. The higher energy level represents the HT-1 state, which was relatively unstable and would evolve into a lower energy state by the decomposition of supersaturated L1_2_ phase. With additional heat treatment at 1,023 K for 20 h, phase decomposition occurred and lead to the formation of metastable FCC particles in the L1_2_ phase at a lower energy level as the HT-2 state. With further ageing at 1,023 K for 200 h, the metastable FCC particles would mostly be eliminated by diffusing FCC formers into the surrounding FCC matrix, eventually, the whole system would reach the equilibrium state.

### High temperature tensile properties

**Figure 4:**
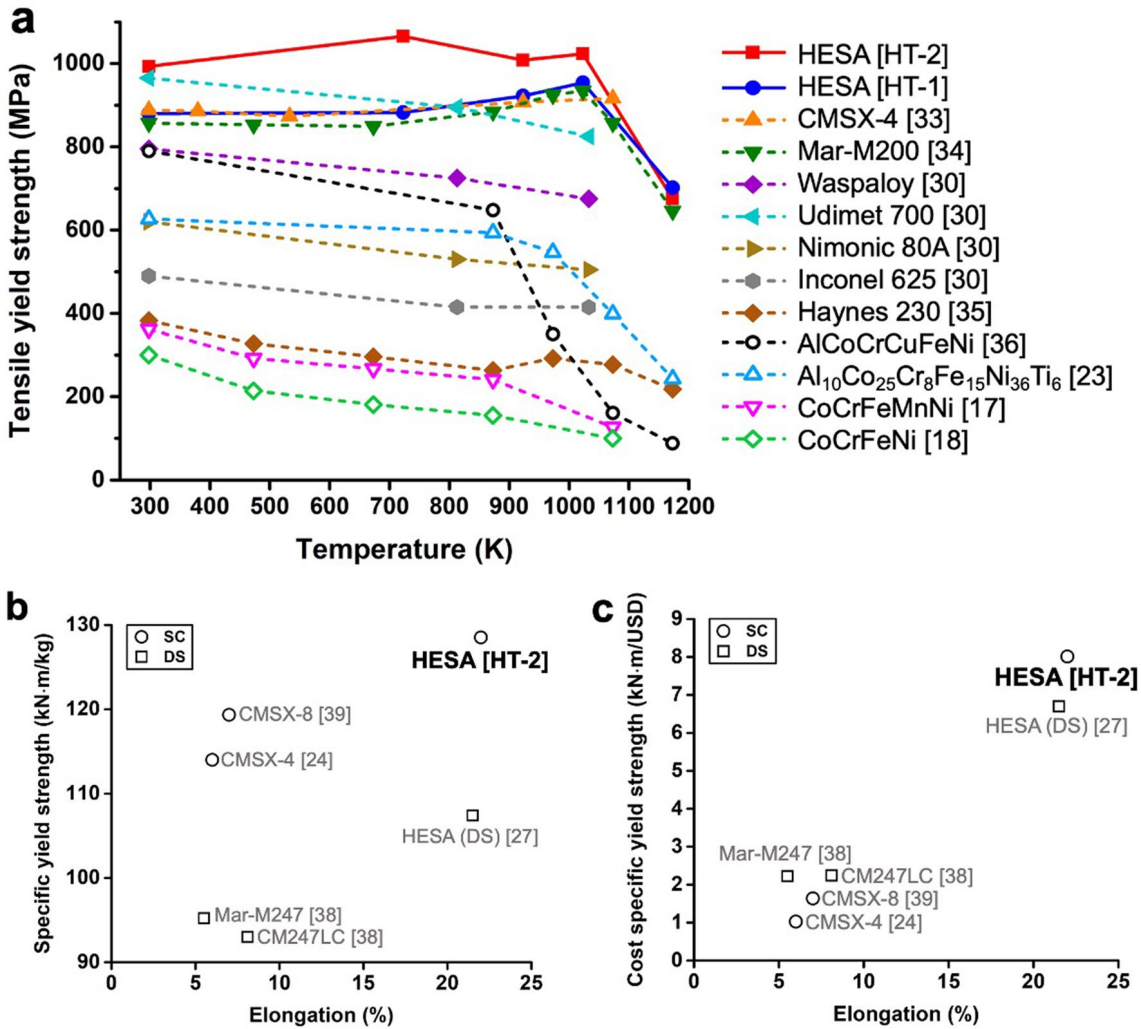
High temperature tensile properties and the combination of high temperature specific yield strength—raw material price—elongation in HESA. (**a**) Tensile yield strength versus temperature, (**b**) Specific yield strength versus elongation at 1,023 K, (**c**) Cost specific yield strength versus elongation at 1,023 K. In (**b**) and (**c**), data of single crystal (SC) alloys and directionally solidified (DS) alloys are marked by circle and square, respectively.

Tensile tests were conducted at 298 K, 723 K, 923 K, 1,023 K and 1,173 K on HESA in HT-1 and HT-2 states, the whole tensile stress–strain curves are presented in Supplementary Fig. S3. Figure 4a shows the tensile yield strength versus temperature plot, which includes HESA, some advanced superalloys^30,33–35^ and some conventional HEAs^17,18,23,36^. The single-phase CoCrFeMnNi HEA possessed a yield strength of 362 MPa at 298 K and 156 MPa at 1,023 K^17^. Al_10_Co_25_Cr_8_Fe_15_Ni_36_Ti_6_ showed a yield strength of 627 MPa at 298 K and 473 MPa at 1,023 K^23^. By contrast, HESA in HT-1 state could achieve a yield strength of 880 MPa at 298 K and 954 MPa at 1,023 K, both were higher than those of CoCrFeMnNi and other HEAs, approaching the yield strength level of advanced superalloy such as CMSX-4 (888 MPa at 298 K and 913 MPa at 1,023 K)^33^. For fair comparison in this work, the data of CMSX-4^33^ were selected from those of as-cast single crystals treated by the standard heat treatments without hot isostatic pressing (solutioned heat treatment at 1549 K/2 h + 1,560 K/2 h + 1569 K/3 h + 1577 K/3 h + 1588 K/2 h + 1594 K/2 h + 1597 K/2 h (air cooling), and two step ageing treatments at 1,353 K/4 h (air cooling) + 1,144 K/20 h (air cooling)). The standard heat treatments of CMSX-4 varied in literatures, so the reported yield strength at 1,023 K could vary as well^24,33,37^; for consistency, the yield strength data of CMSX-4 in Fig. 4 were from the same literature^33^. Impressively, HESA in the HT-2 state exhibited even higher yield strength (993 MPa at 298 K and 1,023 MPa at 1,023 K) comparing to those of the HT-1 state. The yield strength of the HT-2 state has surpassed several advanced superalloys from room temperature to elevated temperatures. At 1,023 K, the yield strength of HESA in HT-2 state was 110 MPa higher than that of CMSX-4^33^. However, when the testing temperature was raised to 1,173 K, yield strength of the HT-1 and HT-2 states became similar.

Figure 4b,c summarize the specific yield strength, cost-specific yield strength and elongation at 1,023 K for HESA and several advanced superalloys^24,27,38,39^. Specific yield strength is the yield strength divided by density, and cost specific yield strength is the specific yield strength divided by the raw material cost. In Fig. 4b, HESA shows an excellent high temperature specific yield strength, which is 11% higher than that of CMSX-4. The CMSX-4 data shown in Fig. 4b,c was taken from previous work by Matsubara et al.^24^, which provided data of the specific tensile yield strength and elongation at 1,023 K. Figure 4c also includes CMSX-8^39^, which is an improved cost-performance version of single crystal superalloy and possesses 1.6 times the cost specific yield strength of CMSX-4. By contrast, HESA presents a remarkable advancement with 8 times the cost specific yield strength of CMSX-4. In addition, at 1,023 K, HESA exhibited a tensile elongation greater than 20%, which was three times greater than that of CMSX-4, Fig. 4b,c. These results suggest HESA as a cheaper, lighter, stronger and more ductile alloy as comparing to advanced superalloys.

**Figure 5:**
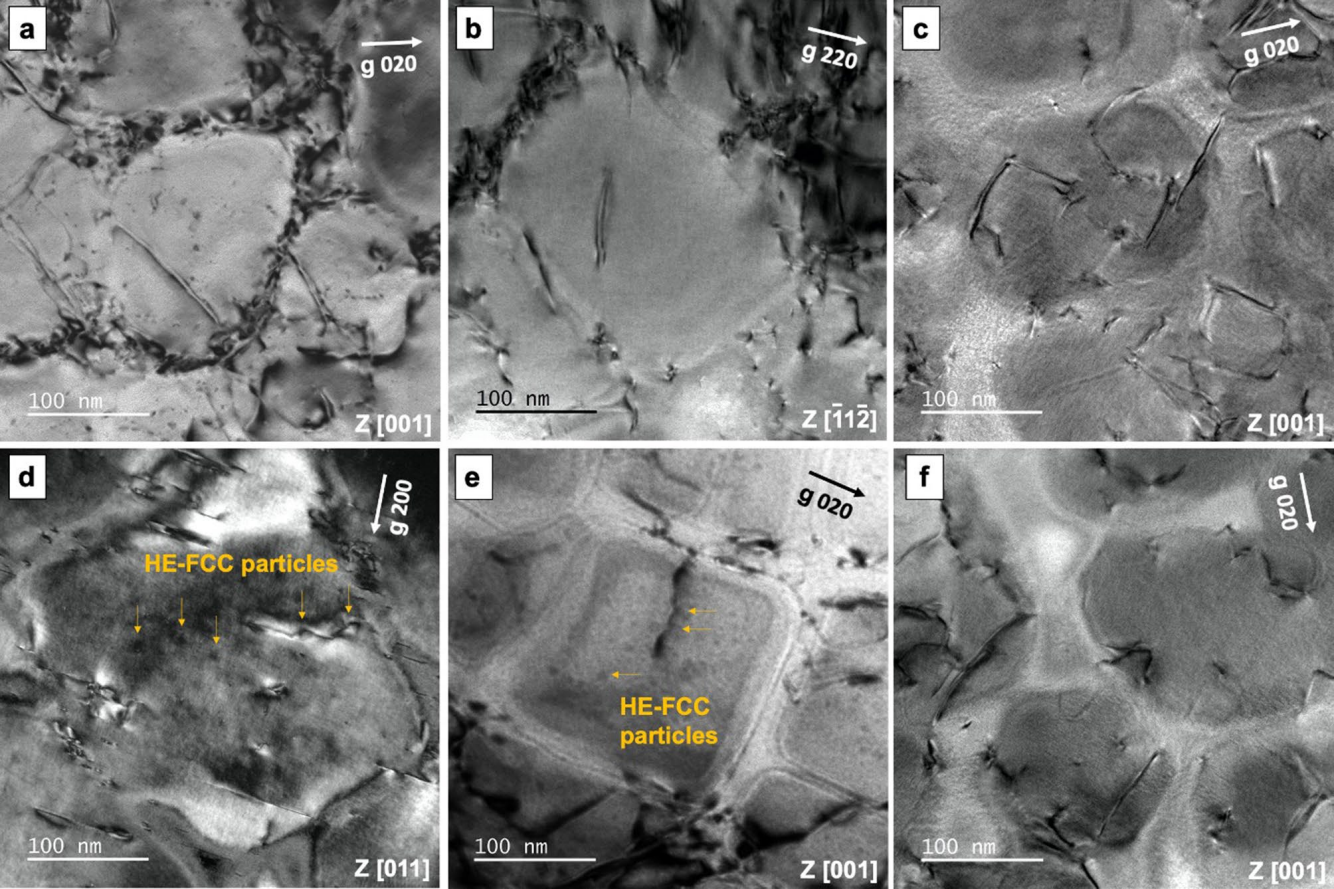
TEM analysis on tensile ruptured samples. HT-1 samples tested at (**a**) 723 K, (**b**) 923 K, and (**c**) 1,173 K, and HT-2 samples tested at (**d**) 723 K, (**e**) 923 K, and (**f**) 1,173 K.

TEM analysis on tensile-tested samples are shown in Fig. 5; HT-1 samples tested under 723 K, 923 K and 1,173 K are shown in Fig. 5a,c, respectively; there were dislocations accumulated at the HE-FCC matrix and ME-L1_2_ precipitates interfaces, and ME-L1_2_ precipitates were sheared by pairs of superdislocations that appeared to be straight and parallel. TEM analysis on HT-2 samples tested under the same conditions are shown in Fig. 5d–f. At 723 K (Fig. 5d) and 923 K (Fig. 5e), pairs of superdislocations in ME-L1_2_ precipitates appeared to be wavy, this indicates that HE-FCC particles could act as obstacles for superdislocations within ME-L1_2_ precipitates. At 1,173 K (Fig. 5f), the HE-FCC particles disappeared, and the yield strength of HESA in the HT-1 and HT-2 states became similar at 1,173 K, Fig. 4a.

## Discussion

There have been several important HEA studies reporting breakthroughs in strength-ductility synergy at cryogenic and room temperatures^7,20–22,40^; however, none of them have addressed the high temperature mechanical properties of these HEAs. Therefore, this article uniquely addresses the issue of high temperature tensile strength of HEAs, especially the HESA in the HT-2 condition has performed exceptionally well comparing reported HEAs and advanced superalloys, Fig. 4. The dispersion of HE-FCC particles in ME-L1_2_ precipitates can pin superdislocations, Fig. 5, attributing to an average increase of 113 MPa in yield strength comparing to that of the HT-1 condition. The distinctiveness of the hierarchical microstructure achieved in this work and the strengthening contribution associated with HE-FCC particles are discussed in this section.

Microstructure observations indicate that minor secondary L1_2_ could be identified in HT-1 and HT-2 conditions, Figs. 1 and 2. In traditional cast superalloys, secondary L1_2_ particles could form during the cooling process after solution heat treatment^19,30,41–43^. And, HT-1 sample also possessed some secondary L1_2_ phase possibly during the air cooling process. Secondary L1_2_ particles appeared to coarsen after HT-2 process, Figs. 1 and 2. Although this microstructure evolution suggested the metastability of the FCC matrix, the amount of secondary L1_2_ particles identified was very minor in both HT-1 and HT-2 states in this work. Previous studies on superalloy have speculated that high fractions of secondary L1_2_ phase may decrease the net width of FCC channel and improve mechanical strength^41^; in this work, secondary L1_2_ fractions were too little to affect mechanical properties. In the future, metastable FCC matrix channel should be explored further by heat treatment design to investigate if additional hierarchical microstructure strengthening can be achieved.

To the best of authors’ knowledge, only few literatures have reported the dispersion of FCC particles in L1_2_ matrix^44–46^. Vogel et al.^44^ studied FCC particles in Ni_78_Al_13_Ti_9_ L1_2_ matrix alloy with the partitioning coefficients of Al (0.6), Ti (0.42) and Ni (1.14); Ni showed preferential partitioning toward FCC phase, and high Ni content in the L1_2_ phase was deduced to be responsible for the FCC particle formation. Meher et al.^45^ designed a Re and Ru bearing Ni-based superalloy, and FCC particle dispersion in L1_2_ phase was found within the dendritic microstructure; the supersaturation of Co, Re and Ru in the L1_2_ phase was suggested to be the driving force for the formation of FCC particles. So, the supersaturation of FCC phase partitioning elements in the L1_2_ phase is the cause for the formation of the FCC particles as shown in Fig. 3. The supersaturation achieved in the L1_2_ phase of HT-1 sample promoted the formation of FCC particles and reduced the degree of supersaturation in the L1_2_ phase of the HT-2 state. The ME-L1_2_ phase in HESA possessed the formula (Ni, Co, Fe, Cr)_3_(Al, Ti, Cr, Nb), which contained more FCC phase partitioning elements and a higher entropy value of 1.38 R than that of the L1_2_ phase in CMSX-4 (1.1 R)^46^. High contents of Co and Fe are reported to narrow the L1_2_ phase field and affect phase boundary gradient^47,48^ between FCC and L1_2_ phases, hence high content of Co and Fe in HESA would promote the supersaturation in the L1_2_ phase during air cooling process of HT-1. By contrast, very minor fractions of FCC particles (< 0.3 vol%) was reported in the L1_2_ phase of CMSX-4^46^, in this work HESA possessed much more nano FCC particles (4.3 vol%) in the ME-L1_2_ phase. So, the high entropy composition of HESA might have contributed to high degree of supersaturation in ME-L1_2_ phase and promoted the formation of relatively high fraction of HE-FCC particles. Moreover, alloying with Co and Fe were found to improve the intrinsic ductility of L1_2_-Ni_3_Al phase^49,50^, which could lead to ductile L1_2_ precipitate strengthened HEAs^20^. Therefore, ME-L1_2_-(Ni, Co, Fe, Cr)_3_(Al, Ti, Cr, Nb) phase may contribute to the outstanding tensile elongation of HESA in additional to the ductile FCC matrix as comparing to that of CMSX-4, Fig. 4b,c.

In a previous work on Ni_86.1_Al_8.5_Ti_5.4_ alloy^44,51,52^, the FCC particles could coarsen into directional plates in the L1_2_ matrix after prolonged ageing at 1,023 K. Meher et al.^45^ also found that ageing at 1,073 K could coarsen the FCC particles and decreased its fraction significantly. In this work, since the FCC particles in the L1_2_ phase were in metastable states, all the FCC particle forming elements would diffuse across the L1_2_ phase into the surrounding FCC matrix after prolonged ageing, so that the whole system could reach the equilibrium state, Fig. 3c. This could also explain the disappearance of HE-FCC particles in HESA at 1,173 K, Fig. 5f. Although thermodynamic process is inevitable during thermal exposure, engineering alloys are rarely designed to be used in thermodynamic equilibrium state. In future work, it might be possible to prolong the rate of dissolution of FCC particles by further heavy elements addition, such as Mo, since Mo possesses low diffusivity and preferentially partition toward FCC phase. Addition of more heavy elements might be able to slow down the evolution of metastable hierarchical microstructure further and prolong its service life for high temperature application.

There is a very limited number of papers reporting benefit of the kind of hierarchical microstructure by experiments^44,45,52–59^. Vogel et al.^44^ showed a 50 HV hardness increase with the presence of FCC precipitates in Ni_78_Al_13_Ti_9_^44^ L1_2_ matrix alloy. In addition, Tian et al.^54^ reported a 78 MPa increase in compression strength at 973 K by the FCC precipitation in Ni_78_Al_18_Ti_4_ L1_2_ matrix alloy. Meher et al.^45^ showed the L1_2_ phase with nano-sized FCC particles could possess enhanced coarsening resistance for the L1_2_ phase. Smith et al.^56^ found that the FCC particles inside L1_2_ phase might increase compression creep life from 20 to 90 h under 1,033 K/414 MPa. Although these studies demonstrated the strengthening contribution of the FCC precipitation in L1_2_ matrix, these were mainly L1_2_ matrix in bulk and could be brittle in tension. Notably, this article is the first to report tensile properties of this kind of hierarchical microstructure in tension, even at elevated temperatures.

To elucidate the outstanding tensile yield strength of HESA, each strengthening factor needs to be examined. The yield strength of HESA in the HT-2 state was 113 MPa higher than that of the HT-1 state. Since the phase compositions of both FCC matrixes and both L1_2_ phases in the HT-1 and HT-2 states (Table 1) were very similar, their difference with respect to anti-phase boundary (APB) energies of L1_2_ phase, stacking fault energies of the FCC matrix, and the lattice misfits should be minimal; this implies that additional strengthening contributions from the intrinsic properties of HE-FCC matrix and ME-L1_2_ phase could be excluded. So, higher strength in the HT-2 state might arise from higher ME-L1_2_ phase volume fraction, larger ME-L1_2_ phase size, and the presence of HE-FCC particles. According to the TEM analysis in Fig. 5b and e, paired dislocations resided within the ME-L1_2_ precipitate. The pair-coupling model^19,27^ could be applied to calculate contributing factors of L1_2_ precipitate sizes and fractions on critical resolved shear stress (CRSS):

1 $$  \tau _{c}  = \sqrt {\frac{3}{2}}  \times \left( {\frac{{Gb_{{fcc}} }}{R}} \right) \times \frac{{\sqrt F \varphi }}{{\pi ^{{\frac{3}{2}}} }} \times \sqrt {\frac{{2\pi R\gamma _{{APB}} }}{{\varphi Gb_{{fcc}}^{2} }} - 1}  $$

where ${\tau }_{c}$ is the CRSS, γ_APB_ is the APB energy, F is the L1_2_ phase volume fraction, R is the L1_2_ phase radius, b_fcc_ is the Burgers vector in the FCC matrix, G is the shear modulus and φ is a dimensionless constant accounts for the elastic repulsion between the paired dislocations. To assist the calculation, the values of γ_APB_, b_fcc_, G and φ were taken from the previous work^27^ and JMatPro calculations^60^ to be 0.22 J/m^2^, 0.257 nm, 81 GPa and 1, respectively. The HT-1 condition contained an average L1_2_ phase size of 143 nm and the L1_2_ phase volume fraction of 68.4%; the HT-2 condition possessed an average L1_2_ phase size of 153 nm and the L1_2_ phase volume fraction of 70.6%. The calculation result indicates that the difference in CRSS between these two states was less than 1%, which means that slight difference in the L1_2_ phase sizes and fractions between HT-1 and HT-2 had very limited impact on yield strength. Furthermore, previous study has shown the limited effect of secondary L1_2_ particles on tensile properties^42^. With only little amount of secondary L1_2_ particles (< 0.5 vol%) found in this study for both HT-1 and HT-2 samples, its influence on strength can be minimal. Therefore, the origin of additional strengthening contribution should be associated with the presence of the HE-FCC particles inside ME-L1_2_ phase at HT-2 state.

Previous studies from Nemoto et al.^55^, Hirsch et al.^59^, Pretorius et al.^61,62^, Liu et al.^63,64^ and Ardell et al.^65^ have deduced the strengthening contribution for ordered matrix by disordered particles; dislocations inside the L1_2_ phase could be attracted to the disordered FCC particles that had no APB penalty and possessed shorter magnitude of burgers vector. This can explain the wavy superdislocations observed by TEM in Fig. 5d,e; to make dislocations wavy is to make it longer, hence energy requirement for dislocation motion would be higher with the presence of HE-FCC particles. In this work, Hirsch’s model^59,65^, was applied to evaluate the strengthening contribution in CRSS as:

2 $${\tau }_{c}=\frac{1.1455K}{r{ b}_{{L1}_{2}}}\times \sqrt{\frac{3f}{4\pi }}\times \sqrt{\frac{r\pi {\gamma }_{APB}}{K}-1}$$

where ${\tau }_{c}$ is the CRSS, r is the average radius of FCC particles, f is the volume fraction of FCC particles in the L1_2_ phase, b_L12_ is the Burgers vector in L1_2_ phase, γ_APB_ is the APB energy and K is the repulsive force between the edge superdislocations which can be stated as:

3 $$K=\frac{G{b}_{fcc}^{2}}{2\pi \left(1-\nu \right)}$$

And, G is the shear modulus, b_fcc_ is the Burgers vector in FCC phase, ν is the Poisson’s ratio. To derive CRSS, the γ_APB_, b_fcc_, b_L12_, ν and G were taken from the previous work^27^ and JMatPro calculations^60^ as 0.22 J/m^2^, 0.257 nm, 0.514 nm, 0.313 and 81 GPa, respectively. Also, the average HE-FCC particle radius and volume fraction were measured as r = 4 nm and f = 4.3%, respectively. The predicted CRSS is 78 MPa, then, the CRSS value should be multiplied by 0.706 to fit the L1_2_ phase fraction in HESA. The estimated increase in yield strength should be two times higher than the CRSS values, since the maximum value of Schmid factor is 0.5. Hence the increase in yield strength due to nano HE-FCC particles is 110 MPa, which is almost the same as the actual yield strength increase determined by experiment, i.e. 113 MPa, from the HT-1 state to the HT-2 state. This reveals the profound strengthening contribution of nano HE-FCC particles inside ME-L1_2_ phase. The calculated data for the increase in yield strength as a function of the average FCC particle radius (Supplementary Fig. S4) shows the 4 nm of particle radius in the HT-2 state was very close to the peak strength of 110.18 MPa with 3.6 nm particle radius.

Hierarchical HESA has shown outstanding high temperature tensile properties in this work. Creep resistance is also an important property for high temperature application. Although the disappearance of FCC particles at 1,023 K after 200 h, Fig. 3c, suggested that HESA would have to rely on remaining L1_2_ precipitates for strengthening, our previous work^27^ has shown that without nano FCC particles, HESA possessed similar creep resistance as that of CMSX-2. The future challenge would be to prolong the thermal stability of FCC particles inside L1_2_ phase, this might be the future direction to develop more creep resistant HESA. Alloy design may utilize more slow diffusing species such as Mo to improve the stability of hierarchical microstructure, and CALPHAD method will need to be utilized for alloy design to avoid formation of detrimental topologically close-packed (TCP) phases^19,30^.

In summary, this work has introduced a novel hierarchical microstructure in HESA, which has the potential to surpass advanced superalloys in terms of tensile properties and cost-performance. HESA can be 8% lighter and 84% cheaper than that of commercial single crystal superalloy CMSX-4; at 1,023 K, the cost specific yield strength of HESA is 8 times that of CMSX-4 while its tensile strain can reach 20%, which is 3 times that of CMSX-4. HESA is strengthened by an interesting hierarchical microstructure consisted of FCC matrix, L1_2_ precipitates inside FCC matrix, and FCC particles inside L1_2_ particles. Superdislocations inside L1_2_ precipitates appeared to be pinned by FCC precipitates, and this could explain why HESA could be stronger than those containing only FCC matrix + L1_2_ precipitates. The high entropy composition of HESA is an important factor for achieving this hierarchical microstructure by phase transformation pathway through metastability. A template for lighter, stronger, cheaper, and more ductile high temperature alloy is proposed.

## Methods

The HESA of interest is Ni_48.3_Co_16.9_Al_10.2_Fe_8.9_Cr_7.4_Ti_5.8_Nb_1.2_Mo_0.9_W_0.4_ (at.%)^27^. The configurational entropy (ΔS_conf._) of the nominal composition is 1.58 R, which agrees the HEA definition (ΔS_conf._ ≥ 1.5 R). HESA samples were fabricated into single crystal bars supplied by the Superalloys and High Temperature Materials Group in National Institute for Materials Science (NIMS), Japan. These single crystal bars were cast by Bridgman process. Laue X-ray method was used to make sure the < 100 > orientation of each single crystal bar. Then, the as-cast single crystal bars were solution heat treated by a single ramp process from room temperature to 1,500 K in 20 h and air cooled to room temperature (this heat treatment process is termed HT-1). After HT-1 process, an additional heat treatment was performed at 1,023 K for 20 h followed by water quench (the entire heat treatment process is termed HT-2). To further examine the microstructure evolution of HESA, a prolonged ageing after HT-1 was done at 1,023 K up to 200 h followed by water quench.

Scanning Electron Microscope (SEM: JEOL 7200F) and Transmission Electron Microscope (TEM: JEOL JEM-F200, 200 kV) were utilized for microstructure characterization. The specimens for SEM observation were polished and electro-etched in the etchant containing 20% H_3_PO_4_ + 80% H_2_O at 2.5 V. Measurement of the phase fraction and size were based on the SEM images. The size and fraction measurements were performed by Nano-Measurer image analysis software and ImageJ software, respectively; at least three SEM images and over one hundred precipitates were measured. As for TEM analysis, specimens were prepared by grinding and twinjet polishing in the solution containing 10% HClO_4_ + 90% C_2_H_5_OH at 30 V and 243 K. Atom Probe Tomography (APT) analysis was carried out by using laser assisted Local Electron Atom Probe (LEAP 5000 XS). APT specimens, along [100] direction, were prepared by a focus ion beam (FIB: Helios 650) system with the standard lift-out procedure. The APT data were collected under a laser mode with laser wavelength 355 nm, laser pulse energy 25 pJ, laser pulse rate 250 kHz, detection rate 1% and specimen temperature 30 K. And, the collected data were reconstructed and analyzed using CAMECA IVAS 3.8.2 software. Measurement of the FCC nano-particle size and fraction were based on the APT data.

High temperature tensile tests were conducted by Shimadzu testing machine. Flat tensile specimens were machined along the < 100 > direction with the gauge length, width and thickness to be 19.6 mm, 2.8 mm and 3 mm, respectively. The specimens were polished and tested at 298 K, 723 K, 923 K, 1,023 K and 1,173 K with a constant strain rate of 10^–3^ s^-1^.

## Supplementary information

Supplementary information

## Data Availability

All data needed to evaluate the conclusions in the paper are present in the paper and/or the Supplementary Materials. The research data of this study is available from the corresponding author A.C.Y. upon reasonable request.
