# Supplementary material for: Hierarchical microstructure strengthening in a single crystal high entropy superalloy
Source: Sci Rep. 2020 Jul 22;10:12163. doi: 10.1038/s41598-020-69257-8 (PMC7376180; doi:10.1038/s41598-020-69257-8)
Supplement: Supplementary file 1 — Supplementary information [file 41598_2020_69257_MOESM1_ESM.pdf]

Hierarchical microstructure strengthening in a single crystal high entropy superalloy

Yung-Ta Chen[1,2], Yao-Jen Chang[1,3], Hideyuki Murakami[2,4], Taisuke Sasaki[5], Kazuhiro Hono[5], Chen-Wei Li[6], Koji Kakehi[6], Jien-Wei Yeh[1,3], An-Chou Yeh[1,3,*]

[1]Department of Materials Science and Engineering, National Tsing Hua University, 101, Sec. 2, Kuang-Fu Road, Hsinchu, 30013, Taiwan.

[2]Research Center for Structural Materials, National Institute for Materials Science, 1-2-1 Sengen, Tsukuba, 305-0047, Japan.

[3]High Entropy Materials Center, National Tsing Hua University, 101, Sec. 2, Kuang-Fu Road, Hsinchu, 30013, Taiwan.

[4]Department of Nanoscience and Nanoengineering, Waseda University, 3-4-1 Okubo, Shinjuku Tokyo, 169-8555, Japan.

[5]Research Center for Magnetic and Spintronic Materials, National Institute for Materials Science, 1-2-1 Sengen, Tsukuba, 305-0047, Japan.

[6]Department of Mechanical Engineering, Tokyo Metropolitan University, 1-1 Minami-osawa, Hachioji-shi, Tokyo, 192-0397, Japan.

*Corresponding author: An-Chou Yeh, yehac@mx.nthu.edu.tw

Supplementary Materials

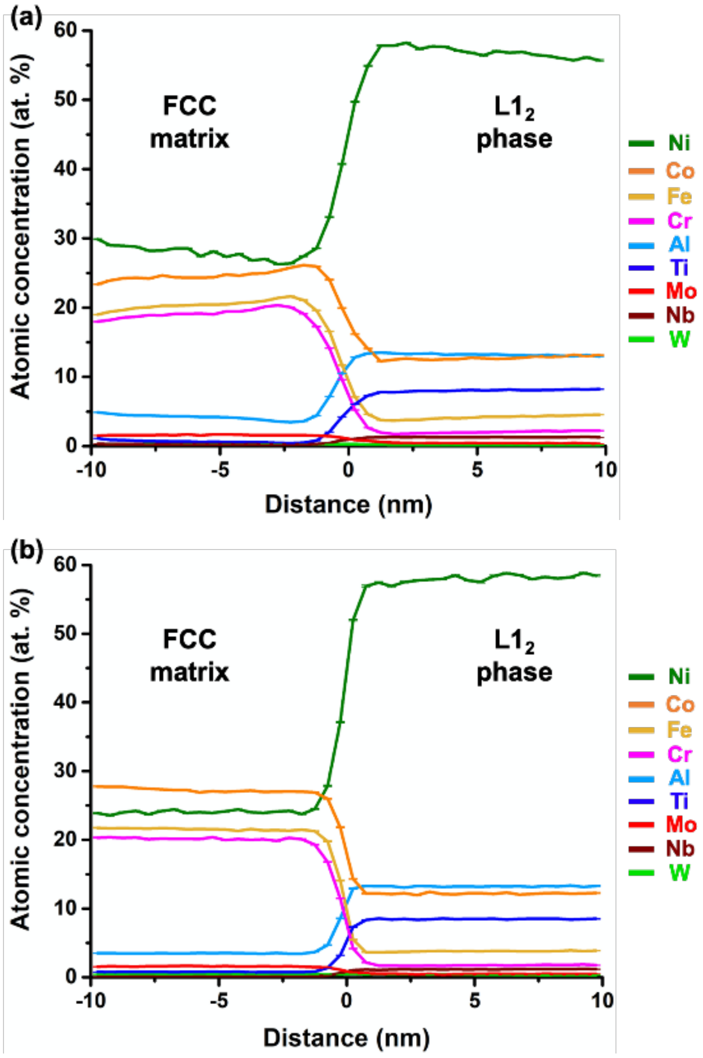

Fig. S1. APT concentration profiles. (a) HT-1 FCC matrix - $L1_2$ phase and **(b)** HT-2 FCC matrix - $L1_2$ phase. The FCC - $L1_2$ phase boundaries are clear in both HT-1 and HT-2 states. Al, Ti, Nb and Ni elements prefer to partition to the $L1_2$ phase; Co, Cr, Fe, Mo and W elements show preferential partitioning behavior toward the FCC phase.

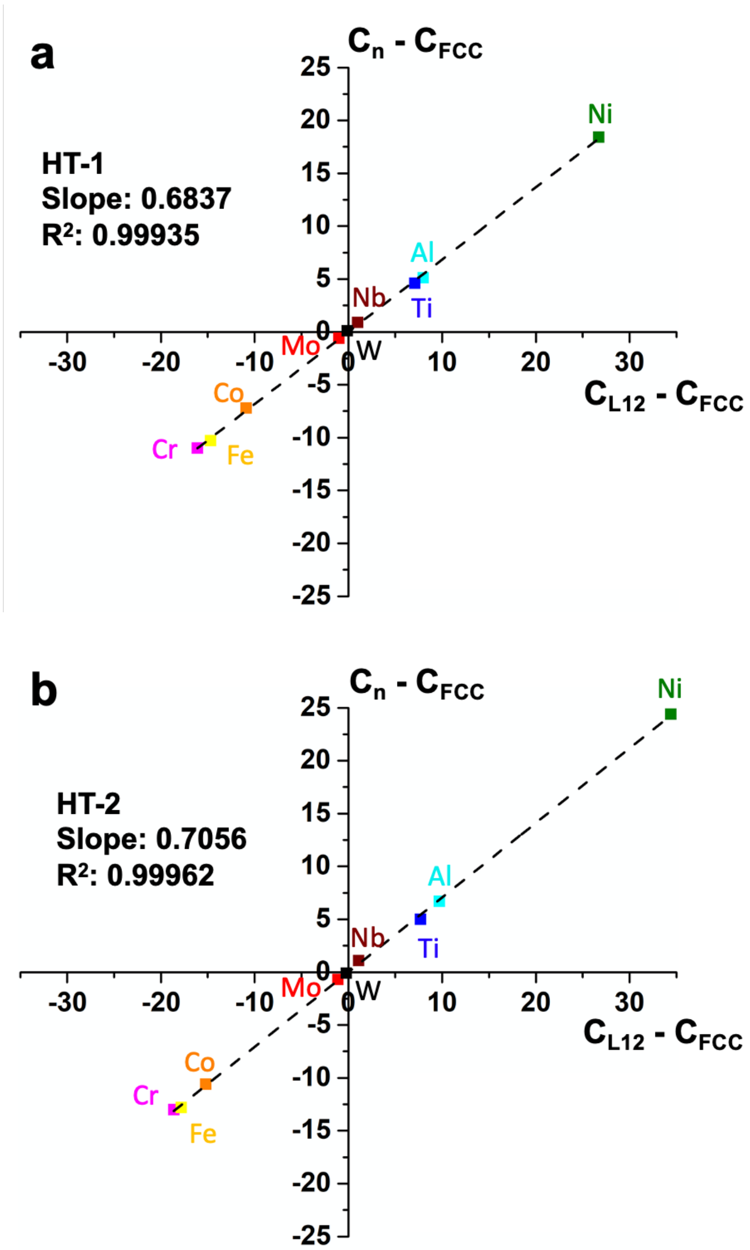

Fig. S2. Lever rule method in confirming $L1_2$ phase fractions. According to the APT composition analysis, plots of $C_{L12} – C_{FCC}$ versus $C_n – C_{FCC}$ from the (a) HT-1 and (b) HT-2 states can be applied to examine the measured compositions. C_{L12}, C_{FCC} and C_n represent the chemical compositions of ME-$L1_2$ phase, HE-FCC matrix and bulk alloy, respectively. And, the slope of the fitting could represent the mole fraction of $L1_2$ phase.

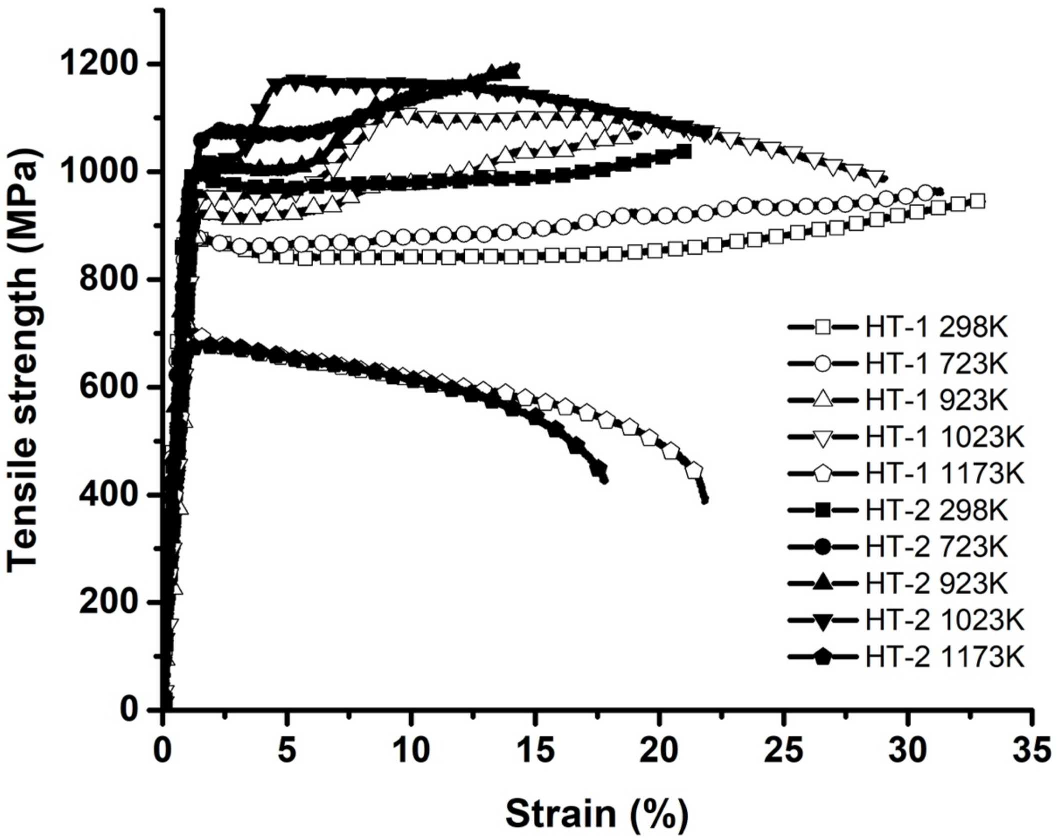

Fig. S3. High temperature tensile strength versus strain of HESA. Tensile tests were conducted at 298 K, 723 K, 923 K, 1023 K and 1173 K on HESA in HT-1 and HT-2 states. HT-1 states are plotted in open symbols; HT-2 states are plotted in solid symbols.

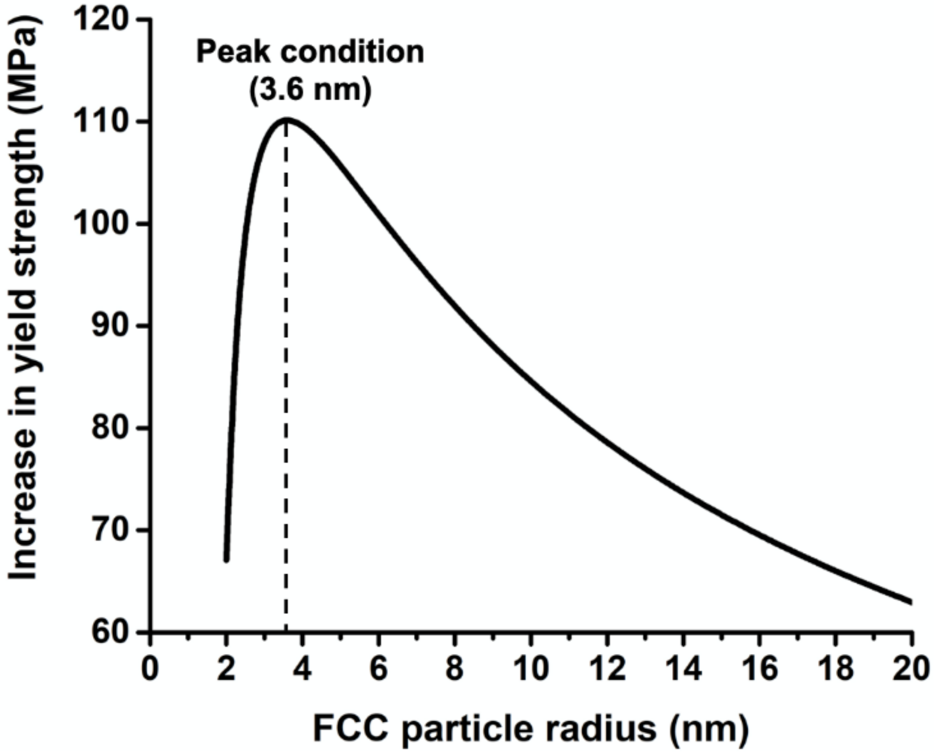

Fig. S4. Plots of increase in yield strength vs. average FCC particle radius. This plot shows the calculation of FCC particle strengthening in HESA by Hirsch's model, and the optimum hardening condition is presented at 3.6 nm.

Table S1. ThermoCalc calculated equilibrium phase concentration at 1023 K

HESA (at. %)		Al	Ti	Nb	Ni	Co	Cr	Fe	Mo	W	$\Delta S_{conf.}$ (-R)
HESA equilibrium at 1023 K	HE FCC matrix	2.6	0.2	0.2	24.8	26.4	20.5	21.9	2.3	1.1	1.61
	ME $L1_2$ phase	14.0	8.6	1.7	59.9	12.2	0.9	2.4	0.2	0.1	1.27
